# Development and Evaluation of Azithromycin-Loaded Transethosomes for Enhanced Dermal Delivery and Antibacterial Efficacy

**DOI:** 10.3390/pharmaceutics17040400

**Published:** 2025-03-21

**Authors:** Meriem Rezigue, Hadeia Mashaqbeh, Alaa A. A. Aljabali, Randa SH. Mansour, Iyad Hamzeh

**Affiliations:** 1 Pharmaceutics and Pharmaceutical Technology Department, Faculty of Pharmacy, Yarmouk University, Irbid 21163, Jordan; h.mashaqbeh@yu.edu.jo; 2 Pharmaceutics and Pharmaceutical Technology Department, Faculty of Pharmacy, Zarqa University, Zarqa 13110, Jordan; rmansour@zu.edu.jo; 3 Faculty of Pharmacy, Jordan University of Science and Technology, Irbid 22110, Jordan; idhamzeh@just.edu.jo

**Keywords:** azithromycin, transethosomes, dermal delivery, antibacterial activity, skin penetration, *Staphylococcus aureus*, vesicular drug delivery, topical treatment

## Abstract

**Background/Objectives**: The topical delivery of antibiotics through transethosomes shows promise for enhancing its dermal delivery for the treatment of skin infections. This study aimed to develop and characterize azithromycin-loaded transethosomes to enhance topical drug delivery and improve the antibacterial activity of azithromycin. **Methods**: The prepared azithromycin formulations underwent assessment for various characteristics, including their vesicle dimensions, size distribution, zeta potential, encapsulation efficiency, and morphological features (via TEM analysis). Additionally, their thermal properties were examined through DSC analysis, and their stability was monitored over six months under refrigerated storage conditions. The sequential tape-stripping technique was employed to conduct ex vivo penetration studies on human skin. Interactions between transethosomes and stratum corneum lipids were examined using attenuated total reflectance Fourier-transform infrared spectroscopy (ATR-FTIR). Additionally, the formulations were tested for their in vitro antibacterial efficacy against *Staphylococcus aureus*. **Results**: The findings revealed that AZ 1 and AZ 2 had vesicle sizes of 108.44 ± 5.72 nm and 70.42 ± 6.02 nm, zeta potential measurements of −11.897 ± 1.820 mV and −34.575 ± 4.535 mV, and high entrapment efficiencies of 99.259 ± 0.086% and 99.560 ± 0.014%, respectively. Transmission electron microscopy (TEM) analysis confirmed the spherical nature of the vesicles, whereas differential scanning calorimetry (DSC) confirmed the successful encapsulation of azithromycin in transethosomes. The formulations exhibited acceptable physical stability at 4 °C for six months. Ex vivo studies revealed a significantly higher deposition of azithromycin in the skin by both transethosome formulations than by the drug solution (*p* < 0.05), with low systemic absorption. Among the formulations, AZ 2 resulted in much deeper skin penetration, with deeper dermal and epidermal layer deposition (1.388 ± 0.242 µg/cm^2^) compared to AZ 1 (four-fold higher, *p* < 0.05) and to the control drug solution (12 times more, *p* < 0.05). Analysis using ATR-FTIR suggested that azithromycin-loaded transethosomes improve the drug penetration by increasing the lipid fluidity and extracting lipids from the stratum corneum. Moreover, the transethosomes loaded with azithromycin demonstrated enhanced antibacterial efficacy against *Staphylococcus aureus*, with minimum inhibitory concentration (MIC) values that were lower than those of the free drug solution. **Conclusion**: The results highlight the promising potential of transethosomes as a novel topical drug delivery system for azithromycin that offers improved therapeutic effects against skin infections

## 1. Introduction

Topical therapy is essential for managing *Staphylococcus aureus* skin infections, which impose a significant major global health burden. Conventional topical treatments frequently encounter difficulties in penetrating the deeper dermal layers, where bacterial colonization persists. This limitation often results in suboptimal therapeutic outcomes and an elevated risk of antimicrobial resistance [1]. Azithromycin, a broad-spectrum azalide antibiotic, demonstrates favorable pharmacokinetic properties and has a proven efficacy against common cutaneous pathogens, including *Staphylococcus aureus*. These characteristics position azithromycin as a promising candidate for use in topical antimicrobial therapy in dermatological applications [2].

First described by Bangham in the 1960s, liposomes are phospholipid vesicles that encapsulate both hydrophilic and lipophilic drugs, but their function is still largely confined to the outer skin layers, rendering their effective penetration into the deeper epidermis and dermis more challenging [3,4]. To overcome the limited skin penetration of conventional liposomes, alternative vesicular systems, such as transfersomes and ethosomes, were developed through modifications in the vesicle ingredients [5]. Transfersomes are innovative surfactant-containing ultra-deformable vesicles that were introduced by Cevc and Blume in 1992 and which allow for flexibility due to their ability to squeeze through intercellular spaces in the stratum corneum [6,7].

Ethosomes, introduced by Touitou et al. in 1997, are vesicles composed of high concentrations of ethanol (20–45%) which improve drug permeation by increasing the lipid fluidization in the skin [8]. Ethosomes have been demonstrated to enhance the skin penetration of active ingredients compared to classical liposomes [9].

Although transfersomes and ethosomes are both significantly more effective than conventional liposomes, they still have limitations [10]. Transfersomes, while highly deformable, do not possess the ethanol-induced permeation-enhancing effect of ethanol, whereas ethosomes, despite their ethanol content, lack sufficient deformability to penetrate deeply.

Introduced in 2012, transethosomes are vesicular carriers composed of phospholipids, edge activators (also known as permeation enhancers), and elevated concentrations of ethanol [11]. Transethosomes combine the advantages of two systems by incorporating edge activators (as in transfersomes) along with ethanol (as in ethosomes), allowing them to deform and permeate the stratum corneum into deeper parts of the skin such as the viable epidermis and dermis [12].

Compared to other vesicular systems, including conventional liposomes, transfersomes, and ethosomes, transethosomes have demonstrated superior penetration and a superior accumulation of numerous drugs in the skin [13,14,15,16,17].

Such an effect grants transethosomes dual activity, enabling them to penetrate more effectively through the stratum corneum while also retaining the drug for an extended period in the skin, which could be particularly beneficial for the use of antibiotics such as azithromycin in treating localized bacterial infections [18,19]. In the case of azithromycin’s delivery, this system offers significant potential to enhance therapeutic outcomes by improving dermal penetration, prolonging drug retention, and maximizing antibacterial efficacy [20].

Commercially available azithromycin primarily exists in a dihydrate crystalline form. This form is obtained when crystallization of the antibiotic occurs in a water/acetone mixture. However, when crystallized in a water/alcohol mixture, azithromycin assumes a monohydrate solid state [21]. According to the Biopharmaceutics Classification System (BCS), azithromycin is a Class II drug with poor aqueous solubility, making dissolution the rate-limiting step in its oral administration. This limitation underscores the need for alternative delivery systems to enhance its bioavailability and therapeutic efficacy. As a result, topical azithromycin presents a promising alternative for the treatment of skin infections [22].

As the body’s largest organ, the skin accounts for 15% of the total body weight and serves as a protective barrier against external factors. It consists of three primary layers: the epidermis, dermis, and hypodermis. The stratum corneum, considered a non-viable epidermis, forms the outermost layer of the skin. Its unique structure is fundamental to the skin’s barrier function, and it is characterized by tightly packed lamellar lipids interspersed within hydrophilic keratin proteins [23]. Bacterial skin infections pose a significant global health challenge that requires prompt treatment. These infections occur when harmful bacteria penetrate both the outer and inner layers of the skin, resulting in either superficial or deep-seated infections [24].

*Staphylococcus aureus*, a bacterium naturally present on the skin of about 30 percent of individuals, is the primary cause of cutaneous infections [25]. Topical antibiotics serve as a first-line strategy for the treatment of skin infections. Nevertheless, insufficient drug concentrations at the site of action can result from poor drug permeation and retention within the skin, necessitating repeated drug applications and frequent administration [1,26]. Extensive research has been conducted on nanoparticles, particularly polymeric and lipid-based nanosystems, to address this challenge. Studies have shown that nanoparticles significantly enhance the performance of antimicrobial agents, thereby reducing the need for frequent use, a key factor contributing to bacterial resistance. These beneficial properties are attributed to the small size, large surface area, and increased reactivity toward pathogens of nanoparticles [27,28,29].

Azithromycin is commercially available in oral, parenteral, and ophthalmic forms. However, to date, there is no registered topical formulation for skin application. Azithromycin typically exists as a dihydrate (log P = 3.98, MW = 785) and has poor aqueous solubility [30]. Incorporating azithromycin into vesicular nanosystems is expected to enhance its solubility in water, provide sustained drug release, and improve its antibacterial effect. Although various studies have explored the topical delivery of azithromycin via liposomes [31], zinc oxide nanoparticles [32], microemulsions [33], and tea tree oil-based nanoemulgels [34] for the treatment of skin infections, these methods have demonstrated limited efficacy due to poor penetration and retention in the skin. Consequently, an improved delivery mechanism remains necessary. Rukavina et al. prepared conventional liposomes, propylene glycol-containing liposomes, and cationic liposomes encapsulating azithromycin. Their findings indicated that all liposomes that they studied significantly improved the skin retention of azithromycin compared to the control [31].

However, to the best of our knowledge, the application of azithromycin-loaded transethosomes has not yet been investigated for topical administration in the treatment of localized skin infections. Compared to other vesicular systems, transethosomes demonstrate superior skin penetration due to their dual mechanism of ethanol-enhanced permeation and edge activator-induced deformability. This combination facilitates enhanced drug delivery, prolonged retention, and increased stability, thereby rendering transethosomes a promising topical delivery system [35].

Consequently, the objective of this research was to formulate azithromycin-loaded transethosomes using the film hydration method, characterize their physicochemical properties, evaluate their ex vivo skin penetration and deposition, and assess their antibacterial efficacy against *Staphylococcus aureus*. We hypothesize that increasing azithromycin retention within the skin will enhance its effectiveness in treating skin infections by reducing the required dosing frequency and limiting overuse, which contributes to bacterial resistance. This research investigated the use of transethosomal nanocarriers to enhance the topical skin delivery of azithromycin.

We examined the physicochemical characteristics of the prepared transethosome formulations, including their vesicle size, size distribution, zeta potential, and encapsulation efficiency, and conducted differential scanning calorimetry (DSC) analysis. Additionally, the stability of these formulations was evaluated. This study assessed the ex vivo skin penetration and deposition behavior of azithromycin-loaded transethosomes using sequential tape stripping and reflectance Fourier-transform infrared (ATR-FTIR) spectroscopy, comparing the results to a control. Moreover, the researchers tested the in vitro antibacterial efficacy of the optimized drug-loaded transethosomes against *Staphylococcus aureus*. While previous studies have explored the use of liposomes, nanoemulgels, and microemulsions for azithromycin delivery, none of these systems possess the combination of deformability and ethanol-enhanced penetration capabilities offered by transethosomes.

## 2. Materials and Methods

### 2.1. Materials

Azithromycin dihydrate was purchased from the Tokyo Chemical Industry (Tokyo, Japan). Clarithromycin was provided by Hikma Pharmaceuticals (Amman, Jordan). Phospholipon 90 G (purity > 94%) was a kind gift from Lipoid GmbH (Ludwigshafen, Germany). The chemicals Tween 80 (polyoxyethylene sorbitan monooleate) and FMOC-Cl (9-fluorenylmethyloxycarbonyl-chloride) were obtained from Sigma-Aldrich (St. Louis, MO, USA). Sodium cholate was sourced from the Tokyo Chemical Industry in Tokyo, Japan. For all other reagents, analytical- or HPLC-grade materials were employed.

### 2.2. Preparation of Azithromycin-Loaded Transethosome Formulations

Azithromycin-loaded transethosomes were prepared using the thin-film hydration technique [36,37]. Briefly, a combination of azithromycin, phospholipid, and surfactant was dissolved in a 1:1 (*v*/*v*) mixture of chloroform and methanol then transferred to a round-bottomed flask. Using a rotary evaporator (Heidolph Rotary Evaporator, Schwabach, Bavaria, Germany), the organic solution was evaporated under vacuum at 40 °C until a thin lipid film formed on the flask’s interior surface. Continuous flask rotation ensured uniform film distribution during evaporation.

To rehydrate the lipid film, a hydroethanolic mixture was introduced. This process occurred at ambient temperature while the flask rotated at 120 rpm. The resulting cloudy suspension was left to swell for 30 min at room temperature, allowing for multilamellar vesicle formation. This step ensured thorough lipid hydration and stable vesicle formation.

To reduce vesicle size and achieve a narrow size distribution, probe sonication was performed using a probe sonicator (Sonics VibraCell, 20 kHz, 50% energy output). To minimize overheating and degradation of the formulation, the sonication was conducted in an ice bath for 5 min with alternating 5 s on/off intervals. The final transethosome suspension was collected and stored in sealed containers at 5 ± 3 °C for later use. The compositions of the transethosome formulations, including azithromycin, phospholipid, surfactant, and ethanol concentrations, are presented in Table 1.

### 2.3. Determination of Vesicular Size, Polydispersity Index, and Zeta Potential

The vesicular size and polydispersity index (PDI) of the prepared transethosome dispersions were analyzed using dynamic light scattering technology on a Malvern NanoZS device (Malvern Instruments Ltd., Malvern, UK) at 25 ± 2 °C. To reduce multiple scattering effects, the samples underwent a 1:10 (*v*/*v*) dilution with deionized water before analysis.

The zeta potential, indicative of vesicle surface charge and dispersion stability, was determined using the same apparatus through laser Doppler anemometry. All experiments were replicated three times, with results expressed as mean values accompanied by their standard deviations (SDs).

### 2.4. Encapsulation Efficiency EE Determination

Unentrapped azithromycin was separated from the transethosome suspension via ultrafiltration in centrifuge tubes (Sartorius Vivaspin, MW cut-off 3000; Sartorius Stedim Biotech Company Ltd., Aubagne, France). Centrifugation was performed at 8000 rpm for 20 min at 4 °C, facilitating thorough separation of the free drug. The unentrapped drug was quantified using HPLC. Entrapment efficiency (EE) was calculated using Equation (1) [38].

(1) $$EE\;(\%)=\frac{\left(AZT-AZF\right)}{AZT}\times 100\%$$

where AZT represents the total amount of drug initially added, and AZF is the amount of unentrapped drug. All experiments were performed in triplicate, and EE values are reported as mean ± standard deviation (SD).

#### HPLC Analysis

For encapsulation efficiency determination, azithromycin was quantified via high-performance liquid chromatography (HPLC) [39]. Analysis was performed using a Shimadzu LC-20A 2030 HPLC system (Kyoto, Japan) with an ACE 5 C8 column (250 mm × 4.6 mm, Avantor^®^, VWR, Radnor, PA, USA) for chromatographic separation.

The mobile phase consisting of 80% methanol and 20% phosphate buffer solution (pH 7.5) was prepared using potassium dihydrogen phosphate and adjusted with sodium hydroxide. The flow rate was set at 1 mL/min, and the column oven temperature was maintained at 40 ± 1 °C. Detection was performed at 210 nm using UV spectroscopy, with a 20 µL injected volume. Under these conditions, azithromycin was eluted at a retention time of 4.5 min. Azithromycin solutions with concentrations ranging from 1 to 25 µg/mL were analyzed to generate a standard calibration curve. The resulting curve demonstrated excellent linearity, with a correlation coefficient of R^2^ = 0.999, validating the method’s reliability for quantitative analysis. To ensure accuracy and reproducibility, all quantitative analyses were performed in triplicates. Azithromycin concentrations in the formulations were determined by comparing sample peak areas to the standard curve.

### 2.5. Transmission Electron Microscopy (TEM) Analysis

The structural features of transethosomes were analyzed using a JEM-1010 transmission electron microscope (JEOL, Tokyo, Japan). Before examination, samples were positively stained with 1% (*v/v*) uranyl acetate. The microscope was operated at an acceleration voltage of 80 kV, with magnification levels ranging from 10,000 to 100,000 times. For TEM analysis, transethosomes loaded with AZ 1 and AZ 2 formulations were selected for evaluation of their morphological characteristics.

### 2.6. Differential Scanning Calorimetry (DSC) Analysis

A differential scanning calorimeter (DSC, Mettler Toledo, Columbus, OH, USA) was used to analyze azithromycin, drug-loaded transethosomes, and unloaded transethosomes. Each sample, weighing approximately 4 mg, was accurately measured and sealed in a standard aluminum pan with a crimped lid to ensure airtight conditions. Samples were heated from 25 to 300 °C, with a steady heating rate of 10 °C/min. A nitrogen purge gas flow of 50 mL/min was maintained to prevent oxidative degradation during the analysis. An empty aluminum pan served as a reference. The resulting thermograms were analyzed to evaluate the thermal properties of the samples, including peak shifts that might indicate drug encapsulation or interactions within the transethosomal system.

### 2.7. Stability Studies

To assess long-term physical stability, transethosomes were stored at 4 ± 2 °C for six months. At specific intervals (0, 1, 2, 3, 4, 5, and 6 months), vesicle size, polydispersity index (PDI), and zeta potential were measured using the methods described in Section 2.3. Each measurement was performed in triplicate to ensure consistency, and results were reported as mean ± standard deviation (SD).

### 2.8. Skin Permeation and Deposition Studies

#### 2.8.1. Preparation of the Skin

Experiments on skin absorption were performed using full-thickness human skin sections acquired from cosmetic surgery procedures. The skin was thoroughly cleansed, and the subcutaneous adipose tissue was removed with a surgical blade. To maintain tissue integrity and remove residual impurities, the cleansed skin samples were rinsed with chilled phosphate-buffered saline (PBS, pH 7.4). Following cleansing, the skin specimens were encased in aluminum foil and stored at −20 ± 2 °C for later use. Before experiments, the skin samples were thawed to ambient temperature and stabilized to ensure uniform experimental conditions.

#### 2.8.2. Ex Vivo Skin Permeation Experiment

Prior to use, human skin samples were immersed in phosphate buffer (pH 7.4) for one hour. Franz diffusion cells (with a 1 cm^2^ diffusion area, Permegear V6A Stirrer, Hellertown, PA, USA) were used to evaluate azithromycin transdermal penetration. The skin samples were trimmed to the appropriate size and positioned with the stratum corneum facing upwards in the donor compartment.

The receptor phase comprised phosphate-buffered saline (PBS, pH 7.4), and the diffusion cells were immersed in a temperature-controlled water bath, maintaining the skin surface temperature at 32 °C. A Teflon-coated magnetic stir bar continuously agitated the receptor compartment. AZ 1 and AZ 2 formulations (1 mL each) were applied to the donor compartment, and drug permeation was evaluated over a 24 h period. A saturated propylene glycol-based drug solution containing the drug served as a control. Each experiment was conducted three times. After 24 h, samples were extracted from the receptor compartment and analyzed for drug content using the method outlined below.

The control drug solution was prepared using propylene glycol as the solvent to maintain azithromycin in a dissolved state. Propylene glycol was selected due to its common use in topical formulations and ability to enhance drug solubility without compromising stability. Although ethanol could have been considered as an alternative solvent, its utilization was avoided due to its presence as a component in transethosomes. Consequently, propylene glycol was selected to ensure a stable, homogeneous control solution for accurate comparative analysis.

#### 2.8.3. Ex Vivo Skin Deposition Experiment

After the 24 h of drug permeation, the skin specimens were extracted and thoroughly cleansed with phosphate buffer-saturated cotton. The stratum corneum was subsequently separated from the underlying viable epidermis and dermis utilizing the tape-stripping method [40]. Azithromycin was extracted from 20 Scotch Book Tape strips (1.5 × 1.5 cm, 3M, St. Paul, MN, USA) by immersing them in acetonitrile. The extraction procedure involved sonication for 30 min, followed by 4 h of agitation at ambient temperature using an Orbi-Shaker (Benchmark, Atkinson, NH, USA).

The remaining viable epidermis and dermis were finely minced and subjected to identical acetonitrile-based extraction procedures. Subsequently, all samples underwent analysis for azithromycin content utilizing pre-column derivatization and fluorescence detection techniques, as delineated in the ensuing section.

To preclude potential interference between the drug peak and endogenous components from the skin or tape, blank skin and tape samples were subjected to the same extraction procedure with acetonitrile and pre-column derivatization.

To ensure consistent stratum corneum removal, uniform pressure was applied during the procedure for each tape strip to standardize the process across all samples. The number of tape strips corresponded with values (15–20) reported in the literature that were previously deemed sufficient for complete stratum corneum removal [41].

The final tape strips were microscopically examined, revealing the presence of nucleated keratinocytes, which indicated the transition from stratum corneum to viable epidermis. This ensured the accurate estimation of azithromycin penetration into deeper skin layers.

#### 2.8.4. Azithromycin Chemical Derivatization and HPLC Analysis

The HPLC method used for encapsulation efficiency determination (Section 2.4) was not sufficiently selective and sensitive to quantify azithromycin extracted from tape strips, skin layers (viable epidermis and dermis), or receiver compartment. Due to weak UV absorption, fluorescent detection required coupling with a fluorescent molecule.

To measure azithromycin extracted during ex vivo permeation and deposition studies, an adapted HPLC technique was employed, incorporating pre-column derivatization and fluorescence detection [42]. The method was optimized for accurately measuring azithromycin in skin samples and the receptor compartment.

Clarithromycin (CLM) was used as an internal standard. Prior to analysis, derivatization was performed using FMOC-Cl (9-fluorenylmethyloxycarbonyl-chloride) as the coupling reagent. Analysis was conducted using a Shimadzu Prominence 2030 HPLC system (Kyoto, Japan) equipped with a fluorescence detector set at 267 nm (excitation) and 317 nm (emission). Data integration was performed using LabSolution Software version 5.98.

Chromatographic separation was achieved on a KYA Japan HIQ Sil C18 column (250 mm × 4.6 mm, 5 µm). The mobile phase consisted of acetonitrile and 0.02 M phosphate buffer (pH 7.7; pH was adjusted to 7.7 using 10% potassium hydroxide) in a 76:24 ratio.

The chromatographic conditions included a flow rate of 1.5 mL/min, an injection volume of 30 µL, and a column temperature of 30 °C.

##### Sample Extraction Procedure

Following the evaporation of acetonitrile, 50 µL of internal standard solution (clarithromycin, 5 µg/mL) was added to each sample. Next, 200 µL of saturated sodium carbonate solution was introduced, and the mixture was vigorously stirred for 1 min to ensure homogeneity. Subsequently, 6 mL of ether was added, and the mixture was vortexed for 2 min. The ether layer was carefully separated and subjected to gentle evaporation under a nitrogen stream at 40 ± 2 °C until fully dried.

##### Derivatization Procedure

The derivatization process was conducted as follows:

After ether evaporation, the remaining residue was reconstituted in 100 µL of acetonitrile and vortex mixed. Next, 100 µL of newly prepared FMOC-Cl solution (1 mg/mL in acetonitrile), 100 µL of water, and 75 µL of phosphate buffer (0.1 M phosphate, pH 7.5) was added. The mixture was incubated in a water bath at 40 °C for 40 min. Upon completion of the derivatization, 30 µL of the final solution was injected into the HPLC system for analysis.

##### Preparation of Calibration Curve

A calibration curve was generated for the extracted samples based on mass quantification. An azithromycin stock solution (100 mg/100 mL) was prepared in acetonitrile. A calibration curve ranging from 0.0005 to 25 µg was established, demonstrating excellent linearity (R^2^ = 0.999). Under these conditions, azithromycin was eluted at a retention time of 11.9 min.

### 2.9. ATR-FTIR Spectroscopy Analysis

The effects of AZ-loaded and unloaded transethosome formulations, as well as the propylene glycol drug solution (control), on human skin were investigated using ATR-FTIR spectroscopy. Ex vivo skin penetration was performed as explained above (Section 2.8.2). After 24 h, the skin specimens were extracted from the Franz diffusion cells and cleansed as previously outlined.

The stratum corneum, which faced the internal reflection element (a ZnSe crystal with a 45° trapezoidal cut), was analyzed using an ATR-FTIR spectrometer (Shimadzu IRAffinity-1S FT-IR Spectrometer, Kyoto, Japan). Uniformity of crystal samples was ensured by applying consistent pressure.

For standardization purposes, a blank was analyzed first. ATR-FTIR spectra were obtained with a spectral resolution of 4 cm^−1^ across the 4000–650 cm^−1^ frequency range. This study focused on the absorbance of C–H symmetric and asymmetric stretching bands at 2850 and 2920 cm^−1^, respectively, and examined shifts in their frequencies. These shifts reflect the disruption of the intercellular lipids organization within the stratum corneum, which is caused by interactions with both drug-loaded and unloaded formulations [40].

### 2.10. Antibacterial Activity

The antimicrobial effect of azithromycin-loaded transethosomes against the gram-positive bacterium *Staphylococcus aureus* was evaluated. For azithromycin-loaded and unloaded formulations, as well as controls, minimum inhibitory concentration (MIC) and minimum bactericidal concentration (MBC) values were determined using the procedure described below.

#### 2.10.1. Inoculum Preparation (Colony Suspension Method)

A disc of *Staphylococcus aureus* strain ATCC 25923 was cultured in 100 mL of tryptic soy broth medium and incubated for 24.0 h at 37.0 °C ± 1.0.

To prepare a fresh (18–24 h) culture plate, a loopful of the broth was streaked onto tryptic soy agar (TSA), a non-selective medium, and incubated at the same temperature. A sterile saline solution was prepared by suspending 3–4 colonies from the organism plate, and the turbidity was adjusted to match a 0.5 McFarland standard using a DensiCHEK© optical device, yielding a bacterial concentration with approximately 1–2 × 10^8^ CFU/mL. The suspension was then diluted to 1:100 by mixing 200 µL of the prepared suspension with 19.8 mL sterile Muller Hinton broth (MHB), producing a 20 mL inoculum containing 1 × 10^6^ CFU/mL.

#### 2.10.2. Broth Microdilution Method

Each sample (drug formulation or control) was introduced directly into the initial well of a 12-well plate by adding 100 microliters of the sample. The remaining wells each received 50 µL of Mueller Hinton broth (MHB). A 1:2 serial dilution was prepared by transferring 50 µL from the first well to the next, which was pre-filled with 1.0 mL of MHB. This process was repeated across nine consecutive dilutions, using fresh pipette tips for each transfer.

Next, 50 µL of the prepared inoculum was added to each well, achieving a final concentration of 5.0 × 10^5^ CFU/mL, which falls within the recommended range of 2–8 × 10 ^5^ CFU/mL.

To verify the inoculum density, an additional 50 µL from each suspension underwent external dilution and culturing. Every plate included a growth control well (containing inoculated broth without the sample) and a negative control well (containing only broth, without sample or bacteria). The plates were then incubated at 37.0 ± 1.0 °C for 24.0 ± 2.0 h.

To determine the MBC, a loopful from MIC wells and higher concentrations was streaked onto Tryptic Soy agar (TSA) plates. These plates were incubated at 37.0 ± °C 1.0 °C for 24.0 ± 2.0 h. After incubation was confirmed by turbid growth in all growth control wells and clear negative control wells, the inoculum density culture results confirmed a concentration of 4–6 × 10^5^ CFU for the tested organisms. All experiments were conducted in triplicate, and MIC and MBC values were determined through visual inspection.

### 2.11. Statistical Analyses

A statistical analysis was performed using Student’s *t*-test with repeated measures. GraphPad Prism 7.0 software was used for data analysis. Results were considered statistically significant when probability values (*p*) were less than 0.05.

## 3. Results and Discussion

### 3.1. Preparation of Azithromycin-Loaded Transethosome Formulations

**Figure 1:**
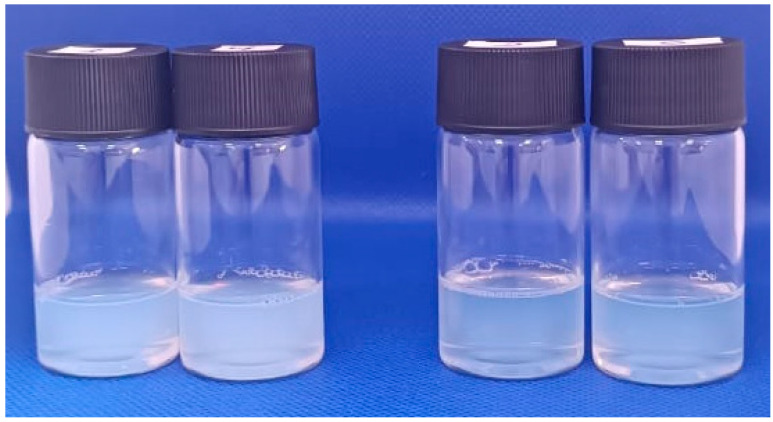
Image for the prepared transethosomes: From left to right: AZ 1, AZ 2, AZ 1 unloaded, AZ 2 unloaded.

We prepared transethosomes using the thin-film hydration technique (Figure 1), which is one of the most widely used methods for creating liposomes. This approach was selected due to its simplicity and the lack of a requirement for sophisticated equipment [43]. Studies have shown that the thin-film hydration technique outperforms the ethanol injection method in the production of luliconazole-loaded transethosomes. Transethosomes prepared using thin-film hydration exhibited vesicular sizes that were one-quarter that of those produced through ethanol injection. Moreover, this method resulted in a formulation that achieved 1.5 times greater drug deposition in the skin compared to its ethanol injection counterpart [44]. These findings highlight the importance of selecting an optimal preparation method to enhance the vesicle properties and improve the efficiency of drug delivery systems.

### 3.2. Determination of Vesicular Size, Polydispersity Index (PDI), Zeta Potential, and Encapsulation Efficiency EE

Table 2 presents the findings for the prepared transethosomes, including measurements of the vesicle dimensions, polydispersity index, zeta potential, and encapsulation efficiency.

The results demonstrate that these formulations exhibit favorable physicochemical attributes for topical drug delivery, including a small vesicle size, uniform size distribution, and high encapsulation capacity.

Particle size plays a crucial role in determining the penetration of a drug through the skin. Smaller particles enhance the contact between the drug and stratum corneum and increase the skin pore size, therefore facilitating the deeper penetration of active ingredients into the dermis [45].

Thus, the vesicular size is crucial when developing nanocarriers for topical and transdermal drug delivery. Studies suggest that a vesicular size of <200 nm is ideal for this route of administration, as it allows drugs to pass through narrow skin pores, resulting in improved skin permeation [17].

As shown in Table 2, the vesicular size of both the loaded and unloaded formulations ranged from 69.41 ± 6.32 nm to 109.77 ± 7.62 nm, with polydispersity index (PDI) values between 0.0663 ± 0.0535 and 0.118 ± 0.021. The formulations exhibited a narrow size distribution, and the PDI values indicated high uniformity in the prepared transethosomes. Similarly to our results, in other studies, minoxidil-loaded transethosomes were in the size range from 69.8 ± 1.4 to 167.4 ± 16.7 nm and sinapic acid-loaded transethosomes were 111.67 nm with a PDI of 0.240 [46,47].

The transethosomes prepared with sodium cholate showed a smaller vesicular size than those prepared using Tween 80 (*p* < 0.05). Similarly, Gillet et al. reported that using a negatively charged surfactant such as sodium cholate minimized the vesicular size of transferosomes by rendering the complex lipid bilayer of this transfersome by increasing the membrane curvature. This effect arises from the interaction between the negatively charged sodium cholate and the positively charged choline group of phospholipids, thereby disrupting the bilayer structure and facilitating curvature formation [48]. Furthermore, sodium cholate is believed to adsorb hydroxyl ions (OH^-^) from water, contributing to the negative charge of transfersomes prepared using this anionic surfactant [49]. The vesicle sizes observed in this study (<200 nm) align with the recommended size range, supporting their suitability for effective dermal drug delivery.

Zeta potential values were assessed to predict the stability of the vesicular systems, as this parameter reflects the degree of repulsion between the nanoparticles. Higher absolute zeta potential values indicate greater electrostatic repulsion, meaning a reduced probability of aggregation and an enhanced formulation stability.

As demonstrated in Table 2, all formulations exhibited negative zeta potential values, which can be attributed to the utilized surfactants and the presence of ethanol [14].

The loaded and unloaded transethosomes prepared using sodium cholate (AZ 2 and AZ 2 unloaded) demonstrated the highest absolute zeta potential values and the smallest vesicle size diameters compared to those prepared using Tween 80 (AZ 1 and AZ 1 unloaded). The pronounced negative charge observed in the sodium cholate-containing transethosomes is attributed to the anionic nature of sodium cholate [50]. Furthermore, the reduced vesicle size observed in these formulations can be attributed to the electrostatic repulsion between highly charged vesicles, which inhibits aggregation.

Although the transethosomes prepared with Tween 80 exhibited lower absolute zeta potential values, previous studies have demonstrated that non-ionic Tween 80 provides steric stabilization rather than electrostatic stabilization, thereby contributing to vesicle stability despite lower zeta potential values [51,52].

The encapsulation efficiency of azithromycin in the loaded transethosomes was high (Table 2), which can be attributed to the lipophilic nature of azithromycin (log P = 3.98). This lipophilicity enhances its affinity for the phospholipid bilayer, particularly within the lipid–drug ratio of 27:1, promoting efficient drug entrapment. These findings elucidate the strong compatibility between azithromycin and the lipid-based matrix of transethosomes, a crucial factor for achieving high encapsulation efficiency. Additionally, the high encapsulation efficiency that was observed may also be attributed to the thin-film hydration method employed in the preparation of the transethosome. In this method, vesicles form over a large surface area within a round-bottom flask, facilitating complete hydration and enhanced drug encapsulation. Similarly, studies have demonstrated that everolimus-loaded transethosomes prepared using the film hydration method exhibited a high encapsulation efficiency which was directly proportional to the lipid–drug ratio [43].

### 3.3. Transmission Electron Microscopy (TEM) Analysis

**Figure 2:**
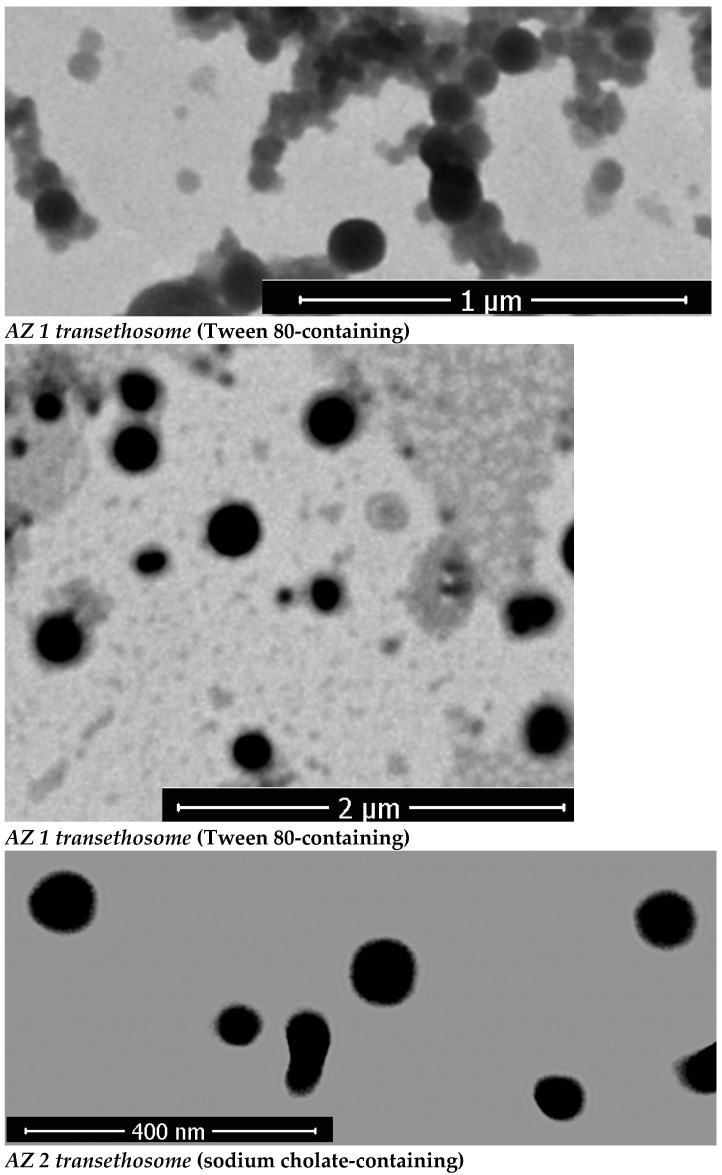
TEM images of the prepared azithromycin-loaded transethosomes AZ 1 (Tween 80-based), AZ 2 (sodium cholate-based).

Figure 2 reports images of azithromycin-loaded transethosomes AZ 1 and AZ 2 visualized by TEM to acquire insight into their morphology. Transethosomes are spherical structures with uniform size distributions and ranges that correlate with those obtained from DLS size measurements (where AZ 1 and AZ2 possess a vesicular size of 108.44 ± 5.72 nm and 70.42 ± 6.02 nm respectively). TEM analysis revealed that the prepared transethosomes exhibit a round shape and consistent size distribution, indicating their structural stability and uniformity. These characteristics further support their potential for use in dermal applications. Other studies have shown similar results in TEM analyses of hexatriacontane-loaded transethosomes [53], paneol-loaded transesthosomes [13], and fisetin-loaded transethosomes [54].

### 3.4. Differential Scanning Calorimetry (DSC) Analysis

**Figure 3:**
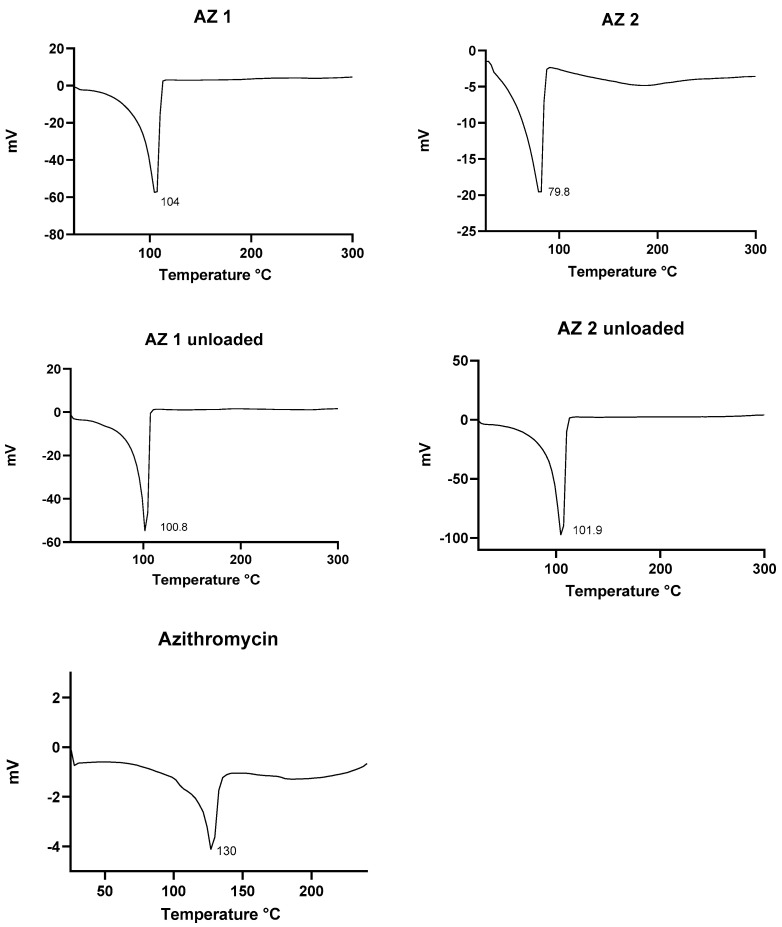
DSC thermograms for the prepared drug-loaded transethosomes (AZ 1, AZ 2), drug-free transethosomes, and azithromycin.

To evaluate the physical state of the vesicular nanosystem, differential scanning calorimetry (DSC) analysis was performed. Figure 3 presents the thermal characteristics of azithromycin dihydrate, the drug-free transethosomes, and the drug-loaded transethosomes. The DSC thermogram of azithromycin’s dihydrate displayed a distinct endothermic peak at 130 °C, consistent with the findings reported by Ghandi et al. This endothermic event corresponds to the dehydration process and may be associated with a recrystallization step in the peritectic transition. The peritectic transition involves the fusion of the dihydrate form, followed by recrystallization of the anhydrous phase into a new crystalline structure [21,55]. In the drug-loaded transethosomes, the absence of the azithromycin endothermic peak suggests that the drug was effectively entrapped within the vesicles and solubilized in the lipid matrix. These findings indicate that the azithromycin was successfully incorporated into the lipid structure of the transethosomal system, potentially enhancing its stability and bioavailability.

Comparable thermal behavior has been observed for propranolol hydrochloride-loaded transethosomes intended for transdermal application by Kumar et al. [56]. Likewise, Mishra et al. confirmed the encapsulation of berberine hydrochloride in transethosomes through DSC analysis [57]. These findings support the potential of this vesicular system as a practical approach for topical drug delivery in the treatment of skin disorders.

### 3.5. Stability Studies

The stability of azithromycin-loaded and unloaded transethosomes was assessed by monitoring changes in their mean vesicular size, PDI, and zeta potential at 4 °C over storage for six months. During this period of transethosome storage, there was no phase separation in any formulation and no drug precipitation. The formulations’ ability to maintain their physicochemical characteristics during refrigerated storage suggests their suitability for extended preservation without degradation. The refrigerated storage of transethosomes at 4 ± 2 °C helps to maintain their vesicle uniformity by lowering the tendency of vesicle fusion, maintaining the integrity and chemical stability of phospholipids, and minimizing drug leakage from the transethosomes during storage; phospholipids exhibit phase transitions at elevated temperatures, and increasing the temperature affects their fluidity and permeability [58,59].

However, the encapsulation efficiency (EE%) was not monitored throughout the storage and, thus, the authors acknowledge that there might have been some degree of undetected drug leakage. Mere visual inspection cannot really guarantee that drug precipitation is non-existent; microscopic or quantitative assessments would provide greater certainty of this point.

In view of these limitations, the vesicle size, polydispersity index (PDI), and zeta potential were studied over a period and could serve as indirect stability indicators. For a period of six months, these variables remained steady, which indicates that there was not much disruption in the vesicles or loss of the drug. Future studies will include EE% studies to verify the long-term stability of the formulations.

The stability study results could show that the azithromycin-loaded transethosomes maintained their physicochemical properties, namely their vesicle size, PDI, and zeta potential, over the period of storage. However, EE% was not determined during the storage period; this would have provided additional information towards fully determining the retention of the drug within the vesicles over time. The measurement of EE% would provide an indication of whether drug leakage (which is more prevalent with hydrophilic drugs) or degradation occurred during storage. Although the above-mentioned studies concluded that transethosomal formulations clearly hold high drug retention abilities within similar conditions, the chance of losing azithromycin is slight. Further studies should ensure EE% measurement at several time points to completely ascertain the stability of the formulation. Furthermore, given that the stability study was conducted at storage conditions (4 °C), further investigation should assess the formulation’s stability at room temperature

**Figure 4:**
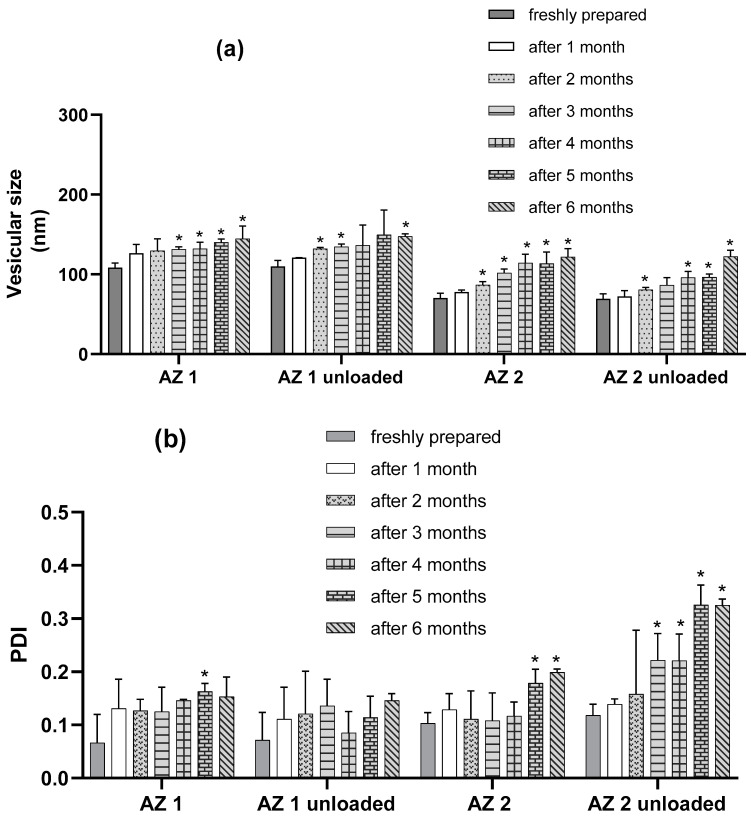

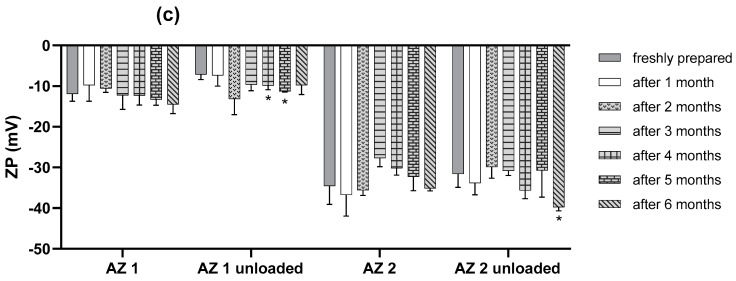
Outline of stability studies of transethosomes over six months at 4 °C in terms of vesicular size (**a**), PDI (**b**), and zeta potential (**c**). (*n* = 3) Data are the mean ± SD. * *p* < 0.05 compared to the freshly prepared formulation.

The results presented in Figure 4 show that the characteristics of the transethosome were not highly altered after six months of storage, although there was a slight increase in vesicular size during storage. However, the PDI values remained lower than 0.2, except for those of the sodium cholate-unloaded formulation, which showed a higher increase in size and PDI than the fresh preparation. The zeta potential values remained nearly unchanged over six months of storage, which proves the good physical stability of the prepared transethosomes.

The results presented in Figure 4 demonstrate that the parameters of all of the transethosomes were not significantly altered after one month of storage (*p* > 0.05). There was no significant modification in the zeta potential values for the loaded formulation during the 6 months (*p* > 0.05). For the loaded formulations, regarding the PDI values, there was no significant modification for the first 4 months of storage (*p* > 0.05) and the PDI was almost lower than 0.2 for the loaded formulations after 6 months, indicating the preservation of the homogeneity of the liposomal population during storage. However, regarding the mean vesicular size after 2 months of storage, there was a significant increase in the mean diameters of AZ 2 compared with their initial values (*p* < 0.05), and, for AZ 1, a significant increase in the mean diameters was observed after the three months of storage (*p* < 0.05). However, the mean vesicular size cannot be evaluated separately from the PDI. Therefore, the prepared tranesethosomes presented an acceptable stability during storage conditions.

### 3.6. Skin Permeation and Deposition Studies

Our research evaluated skin permeation using a method of successive tape stripping.

The findings of the tape stripping study revealed that the azithromycin-loaded transethosomes demonstrated a significantly higher deposition in deeper skin layers when compared to the control. To entirely remove the stratum corneum, a standardized tape stripping protocol was adhered to which precisely controlled the application of pressure, thus allowing an inspection of the final tape strips under the microscope to observe the nucleated keratinocytes. Such validation further confirmed that the measurements of the drug penetration portrayed the true deposition within the viable epidermis and dermis, thus guaranteeing reliability.

**Figure 5:**
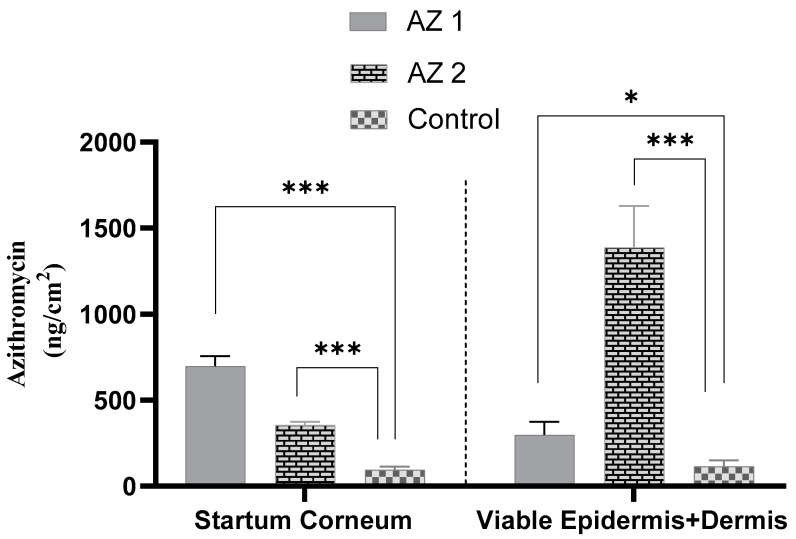
Azithromycin accumulation was assessed in both the stratum corneum and the underlying skin layers (viable epidermis plus dermis) after tape stripping removed the stratum corneum. This evaluation was conducted for transethosomes (AZ 1: tween-based, AZ 2: sodium cholate-based) and a control drug solution (* *p* < 0.05, *** *p* < 0.001) (*n* = 3).

To achieve effective topical antibiotic treatment, it is crucial for the active components to traverse the stratum corneum and infiltrate the deeper skin layers, including the living epidermis and dermis [60]. The distribution of azithromycin in the stratum corneum and remaining skin layers (viable epidermis and dermis) 24 h after applying transethosomes and the control to human skin is illustrated in Figure 5. The transethosome labelled AZ 2 demonstrated the highest concentration of azithromycin in the viable epidermis and dermis (1.388 ± 0.242 µg/cm^2^), followed by the AZ 1 transethosome (0.298 ± 0.077 µg/cm^2^) and, lastly, the saturated propylene glycol drug solution (0.116 ± 0.034 µg/cm^2^) (*p* < 0.05). These findings underscore the enhanced capacity of sodium cholate-containing transethosomes to penetrate the stratum corneum barrier and transport azithromycin to deeper skin regions, where it can effectively exert its therapeutic action.

The in vitro skin deposition of azithromycin by all transethosomes was higher than that of the propylene glycol-saturated drug solution (*p* ˂ 0.05).

The azithromycin-loaded transethosomes and the drug solution control showed a low amount of drug in the receptor phase after the 24 h drug permeation experiment (Table 3). The high sensitivity of the developed HPLC method enables the detection of low drug levels. It can be concluded that azithromycin was deposited and remained mainly localized within its target site, the viable epidermis and dermis, with a low transdermal effect and low systemic absorption.

In our study, azithromycin bypassed the stratum corneum and reached the target site of treatment presented by the viable epidermis and dermis. Therefore, the formulation that provided the highest azithromycin accumulation in these layers was the AZ 2-coded transethosome, which was expected to have the highest therapeutic effect within the skin. The AZ 2 formulation, which incorporated sodium cholate, demonstrated superior skin penetration compared to the AZ 1 formulation, which used Tween 80 instead. AZ 2 achieved a four-fold increase in drug accumulation within the viable epidermis and dermis relative to AZ 1, and a twelve-fold increase compared to the propylene glycol drug solution (*p* < 0.05). Despite the use of AZ 2, which had sodium cholate as an anionic surfactant, not resulting in the highest amount of drug within the stratum corneum, it nevertheless exhibited a higher azithromycin deposition in the viable epidermis and dermis compared to AZ 1, that contained Tween 80, a non-ionic surfactant. This confirms that AZ 2 penetrated throughout the outer skin barrier and into the inner layers of the skin, where azithromycin is effective. The enhanced permeation of AZ2 is induced by two main factors, namely (1) its smaller vesicle size and (2) its higher negative surface charge. The anionic nature of sodium cholate very probably exerted a bigger electrostatic repulsion between the vesicles, causing smaller vesicle sizes, which in turn was expected to increase the penetration into deep skin layers. Further, when compared to neutral or positively charged vesicular systems, negatively charged vesicular systems were previously known to deliver drugs through a much better flux of drug across skin. Our observations support those by Miatmoko et al., who showed that sodium cholate-based transfersomes exhibited a diminished size of vesicles with better dermal penetration than a Tween 80-based formulation [61]. Also, in the research conducted by Gillet et al., the skin penetration of betamethasone and betamethasone dipropionate was significantly increased with negatively charged liposomes when compared to the same using neutral liposomes [48]. In light of these findings, these results imply that sodium cholate, through optimizing the vesicle size and surface charge, gives better transethosomal penetration, hence providing deeper skin layer delivery.

Transethosomal carrier phospholipids, surfactants, and ethanol work together to enhance drug penetration through the skin lipids, which increases vesicle malleability and allows more transethosomes to reach the deep skin layers and release more drugs [11,17,19].

The transethosomes significantly improved the azithromycin delivery compared to the control. Ethanol was introduced into the system to alter the intercellular region of the stratum corneum and to soften the lipid bilayer. As a result, the transethosomes were found to increase the drug accumulation in the epidermal and dermal layers. The postulated mechanisms that explain the increase in azithromycin transport across the skin resulting from its encapsulation into this vesicular system include modifying the skin hydration and improving drug thermodynamic activity [35,62].

During the deposition study, the results showed that the transethosomes encapsulating azithromycin significantly enhanced the penetration of the drug compared to the control drug solution. The control formulation used propylene glycol as the solvent, which was carefully selected to ensure uniform drug dissolution over the course of the study. Although the outer phase of transethosomes contains ethanol, we acknowledge that their slight composition differences in solvents may cause the permeation results to vary slightly. Nevertheless, the remarkable increase in azithromycin penetration shown by transethosomes is strongly suggestive of their enhanced permeation ability, regardless of the solvent effects.

### 3.7. ATR-FTIR Spectroscopy Analysis

**Figure 6:**
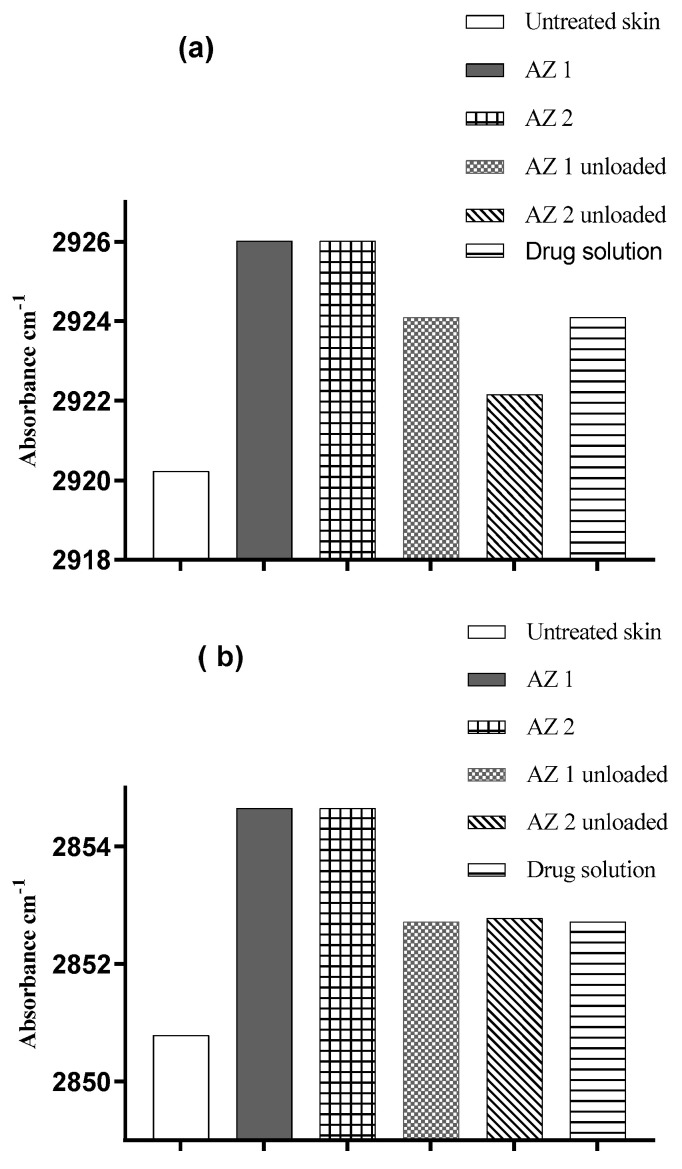
Changes in human skin lipid peak positions for C–H asymmetric (**a**) and symmetric (**b**) stretching absorbances resulting from the application of azithromycin-loaded transethosomes and control substances.

The stratum corneum’s characteristics and molecular structure are commonly investigated using ATR-FTIR spectroscopy, a popular biophysical technique [63]. Researchers have employed this instrument to examine how various formulations chemically affect the structural arrangement of lipids and proteins in the skin. The vibrations of lipids in the stratum corneum are characterized by their asymmetric and symmetric C–H stretching modes, occurring at 2920 cm^−1^ and 2850 cm^−1^, respectively [40]. The impact of the prepared transethosomes and control substances on the frequencies of the C–H symmetric and asymmetric absorbances is illustrated in Figure 6 and summarized in Table 4.

The absorption of symmetric and asymmetric C–H bands at 2850 and 2920 cm^−1^, respectively, can be influenced by increasing disorder in lipid alkyl chains. This disorder may result in a blue shift, causing the bands to move toward higher wavelengths [63].

Compared to untreated skin, all of the azithromycin-loaded and unloaded transethosomes and drug solutions induced an increase (blue shift) in the C–H symmetric and asymmetric stretching frequency bands. The blue shifts detected in the FTIR spectra demonstrate that transethosomes effectively compromise the stratum corneum’s lipid bilayer structure. The alteration in the membrane structure enhances the penetration of drugs by increasing the fluidity of the membrane and promoting the extraction of lipids from the skin’s protective barrier. For the untreated skin samples, the symmetric stretching vibrations were observed at 2850.79 ± 0.4 cm^−1^, while the application of AZ 1 and AZ 2 resulted in a shift towards higher frequencies (blue shift) in the peak position of the C–H symmetric stretching vibrations (2854.63 ± 0.02 cm^−1^ and 2854.65 ± 0.2 cm^−1^, respectively). The asymmetric stretching vibrations shifted from 2920.23 ± 0.2 cm^−1^ for the untreated control skin to 2926.02 ± 0.3 cm^−1^ and 2926.01 ± 0.2 cm^−1^ for the AZ 1 and AZ 2 formulation, respectively. These results indicate the disordering of the intercellular lipids of the stratum corneum and agree with the results of the in vitro drug penetration studies. These increased frequencies are likely caused by the fluidization of the lipidic hydrocarbon chain of the stratum corneum. In conclusion, the FTIR data demonstrated that transethosome formulations containing phospholipids, ethanol, and surfactants improved the drug penetration into the skin, primarily by increasing the fluidity and extracting the lipid bilayer of the stratum corneum [64].

### 3.8. Antibacterial Activity

The in vitro antibacterial efficacy of azithromycin-loaded transethosomes was tested against *S. aureus* ATCC 25923. Both formulations demonstrated comparable antibacterial effects. The findings suggest that incorporating azithromycin into transethosomes enhances its antimicrobial potency, underscoring the promise of this method for treating bacterial skin infections. Table 5 presents the outcomes of this evaluation.

The in vitro antibacterial activity of azithromycin against *S. aureus* was improved when it was encapsulated in transethosomes compared to that of the free drug solution (30% ethanol/water). The azithromycin-loaded transethosomes (AZ 1 and AZ 2) showed enhanced antibacterial activity against *S. aureus*, yielding an MIC value that was two folds lower than that obtained using free azithromycin. Both formulations, AZ 1 and AZ 2, exhibited similar antibacterial activity. Furthermore, the bactericidal activity of the transethosomes was similar to that of the free drug. The enhanced in vitro antimicrobial activity of the azithromycin was independent of the transethosome composition. We were unable to detect the MIC and MBC values for empty transethosomes or hydroethanolic solutions (Table 5).

The observed in vitro antibacterial activity of the azithromycin transethosomes agrees with that obtained by other studies of azithromycin liposomes, which showed a higher activity of liposomal azithromycin in comparison to the free drug against various species of bacteria. Rukavina et al. found that azithromycin liposomes yielded MIC values lower than those obtained with the free drug against methicillin-resistant *Staphylococcus aureus* MRSA [31]. In another study, azithromycin-loaded liposomes presented MIC values that were significantly lower than those obtained with the free drug against *Pseudomonas aeruginosa* strains [65].

Furthermore, Oh et al. prepared azithromycin-loaded liposomes, which presented increased antimycobacterial activity with an increasing effectiveness parallel to the moles’ percentage of negatively charged phospholipids. Azithromycin-loaded liposomes prepared using distearoylphosphatidylglycerol phospholipid (DSPG) and cholesterol at a ratio of 10:5 showed 41-fold more inhibition of intracellular *Mycobacterium avium* growth than free azithromycin. Furthermore, it was observed that ciprofloxacin-loaded liposomes with a negative surface charge were better than neutral liposomes in terms of both cellular uptake and the antibacterial potential of the antibiotic, as these anionic vesicles can enhance bacterial membrane interaction [66]. Therefore, in our study, the observed enhanced antimicrobial activity could be related to the higher drug delivery efficiency of the prepared transethosomes and their negative surface charge. Furthermore, drug encapsulation in transethosomes could augment the antibacterial efficacy of drugs by enhancing drug retention and penetration into bacterial cells and cellular uptake.

With respect to systemic absorption, our findings imply that the unwanted systemic absorption was not substantial. The employed HPLC method’s sensitivities were so pronounced that systemic drug levels were not appreciably detected, indicating that the formulation stays localized within the skin layers. Therefore, future studies in this regard will entail pharmacokinetics studies to fully investigate the quantified systemic levels of the drug after its application to the skin. Although the storage stability was determined at six months, we do realize that, gradually, over time, the vesicle size increased. This size increase could influence the drug retention and penetration efficiency over longer time frames. Therefore, beyond six months of investigation, future studies will focus more on stability while optimizing formulation parameters to limit particle growth, followed by the modulation of drug release and efficacy. Regarding batch-to-batch variability and large-scale production, we recognize that the transethosome formulations used herein perhaps present challenges regarding scalability, reproducibility, and consistency in manufacturing. The variation in vesicle size and encapsulation efficiency is dependent on the preparation conditions; hence, optimization of the process should be carried out to ensure uniformity in across batches. Future studies will focus on the scalability of the preparation method and other process control interventions to ensure the reproducibility of the formulation.

In assessing transethosomal formulations designed for topical application, the development aspects that affect the scope of biocompatibility and safety are equally important. While this study focused entirely on the physicochemical properties of the formulations and their antibacterial efficacy, the potential of ethanol and surfactants to cause irritation ought to have been addressed. Other studies have shown that, in vesicular systems, ethanol does not disrupt the skin integrity or lead to irritation, as long as the concentrations do not exceed 30% in topical formulations [67]. In addition, some biocompatible surfactants like Tween 80 and sodium cholate are used in dermal delivery systems and show negligible irritation [61,68]. In this work, however, direct cytotoxicity or hemolysis studies were not performed. Future studies will include MTT assays for cell viability and in vivo skin irritation models for an exhaustive evaluation of the biocompatibility of azithromycin-loaded transethosomes and their safety for clinical applications.

## 4. Conclusions

According to our current understanding, this research represents the initial exploration of the incorporation of azithromycin into transethosomes for combating skin infections. The topical application of antibiotics is typically the primary approach for treating skin infections. Nevertheless, the limited penetration of drugs into the skin and their restricted retention in the skin result in insufficient drug concentrations at the target site. Furthermore, conducting research on azithromycin for topical skin application is challenging due to the absence of approved topical formulations.

In this study, azithromycin-loaded transethosomes were successfully developed using a combination of phospholipids, edge activators, and ethanol. The formulations underwent extensive evaluation, including assessments of their vesicle size, size distribution, zeta potential, entrapment efficiency, morphology (through TEM analysis), thermal characteristics (via DSC analysis), and stability over a six-month period under refrigerated conditions. The findings revealed that transethosomal formulations significantly improved the ex vivo drug deposition in human skin compared to a free drug solution. Furthermore, these formulations demonstrated enhanced in vitro antimicrobial efficacy against *Staphylococcus aureus*, with MIC values that were lower than that of unencapsulated azithromycin while, reserving the drug’s antibactericidal effect. These results support the potential of transethosomes as an effective delivery system to enhance the topical application of azithromycin.

The effective creation of these formulations marks a notable breakthrough in surmounting the protective barrier of the skin and improving targeted drug administration. This approach offers a promising method for combating skin infections caused by resistant microorganisms such as *Staphylococcus aureus*.

Our future research will focus on integrating azithromycin-loaded transethosomes into a semi-solid medium to achieve an appropriate viscosity for practical skin use. We will optimize this formulation to enhance patient adherence and its user-friendliness while preserving its stability and effectiveness. Furthermore, we intend to assess the safety and efficacy of this new formulation using an animal model, which will generate essential preclinical data to support future translational investigations.

This work highlighted the potential use of transethosomal nanocarriers in improving the topical delivery of azithromycin, as they exhibit better skin penetration and enhanced antibacterial efficacy. Although this work has carried out formulation development and ex vivo evaluation, more efforts must be made to study the in vivo pharmacokinetics, systemic absorption, and drug retention of these formulations after their topical application. Moreover, studying these formulations against resistant strains like MRSA will bring more understanding of their efficacy against multidrug-resistant infections. Stability studies over a longer time period need to be carried out to prove the continued antibacterial efficacy and safety of these formulations for clinical applications. The results of these additional studies are expected to verify the therapeutic relevance of azithromycin-loaded transethosomes for the topical management of bacterial skin infections.

In summary, this investigation underscores the promise of transethosomes as an adaptable and cutting-edge platform for topical drug administration, laying the groundwork for future studies that explore their application in various dermatological disorders.

## Figures and Tables

**Table 1:** The compositions of the transethosome formulations.

Formulation Code	Azithromycin Dihydrate (%, *w*/*v*)	Phospholipid (%, *w*/*v*)	Surfactant (%, *w*/*v*)	Hydration Medium
AZ 1 loaded	0.15	4	Tween 80: 1	%30 EtOH/water
AZ 1 unloaded	0	4	Tween 80: 1	%30 EtOH/water
AZ 2 loaded	0.15	4	Sodium cholate: 1	%30 EtOH/water
AZ 2 unloaded	0	4	Sodium cholate: 1	%30 EtOH/water

**Table 2:** Vesicular size, polydispersity index, zeta potential, and encapsulation efficiency results for the prepared transethosomes AZ 1 (Tween 80-based) and AZ 2 (Sodium cholate-based) (*n* = 3).

Formulation Code	Vesicular Size (nm)	PDI	Zeta Potential (mV)	Encapsulation Efficiency (%)
AZ 1	108.44 ± 5.72	0.0663 ± 0.0535	−11.897 ± 1.820	99.259 ± 0.086
AZ 2	70.42 ± 6.02	0.103 ± 0.020	−34.575 ± 4.535	99.560 ± 0.014
AZ 1 unloaded	109.77 ± 7.62	0.0713 ± 0.0523	−7.155 ± 1.233	/
AZ 2 unloaded	69.41 ± 6.32	0.118 ± 0.021	−31.6 ± 3.291	/

**Table 3:** Drug permeation results after 24 h in vitro skin permeation experiment (*n* = 3).

Formulation Code	Drug Concentration in the Receiver (ng/mL) After 24	Drug Concentration in the Receiver (% Permeated) After 24
AZ 1	79 ± 35	0.005267 ± 0.002333
AZ 2	87 ± 40	0.0058 ± 0.002667
Drug solution	68 ± 20	0.004533 ± 0.001333

**Table 4:** Shifts in maximum absorption of human skin lipids in C–H asymmetric and symmetric stretching bands resulting from the application of azithromycin-loaded transethosomes (AZ 1 and AZ 2) and controls (*n* = 3).

	Asymmetric C–H Stretching (cm^−1^)	Symmetric C–H Stretching (cm^−1^)
Control skin	2920.23 ± 0.2	2850.79 ± 0.4
AZ 1	2926.02 ± 0.3	2854.63 ± 0.02
AZ 2	2926.01 ± 0.4	2854.65 ± 0.2
AZ 1 unloaded	2924.09 ± 0.06	2852.72 ± 0.3
AZ 2 unloaded	2922.16 ± 0.02	2852.78 ± 0.6
Drug solution	2924.09 ± 0.3	2852.72 ± 0.4

**Table 5:** In vitro antibacterial activity of azithromycin-loaded transethosomes against *S. aureus* ATCC 25923.

Formulation Code	MIC	MBC
AZ 1	1.5 (µg/mL)	6 (µg/mL)
AZ 2	1.5 (µg/mL)	6 (µg/mL)
AZ 1 unloaded	>50%	>50%
AZ 2 unloaded	>50%	>50%
Free azithromycin	3 (µg/mL)	6 (µg/mL)
30% Ethanol/water	>50%	>50%

## Data Availability

The data supporting the findings of this study are available from the corresponding author upon request.

## References

[1] Dallo M., Patel K., Hebert A.A. (2023). Topical antibiotic treatment in dermatology. Antibiotics.

[2] Gui Z., Wang H., Ding T., Zhu W., Zhuang X., Chu W. (2014). Azithromycin reduces the production of α-hemolysin and biofilm formation in Staphylococcus aureus. Indian J. Microbiol..

[3] Sharma V.K., Agrawal M.K. (2021). A historical perspective of liposomes-a bio nanomaterial. Mater. Today Proc..

[4] Watts G. (2010). Alec Douglas Bangham. Lancet.

[5] Akl M.A., Eldeen M.A., Kassem A.M. (2024). Beyond Skin Deep: Phospholipid-Based Nanovesicles as Game-Changers in Transdermal Drug Delivery. AAPS PharmSciTech.

[6] El Maghraby G.M., Dragicevic N., Maibach H.I. (2016). Ultradeformable Vesicles as Skin Drug Delivery Systems: Mechanisms of Action. Percutaneous Penetration Enhancers Chemical Methods in Penetration Enhancement: Nanocarriers.

[7] Cevc G., Blume G. (1992). Lipid vesicles penetrate into intact skin owing to the transdermal osmotic gradients and hydration force. Biochim. Et Biophys. Acta (BBA)-Biomembr..

[8] Verma P., Pathak K. (2010). Therapeutic and cosmeceutical potential of ethosomes: An overview. J. Adv. Pharm. Technol. Res..

[9] Touitou E., Godin B., Weiss C. (2000). Enhanced delivery of drugs into and across the skin by ethosomal carriers. Drug Dev. Res..

[10] Garg V., Singh H., Bimbrawh S., Singh S.K., Gulati M., Vaidya Y., Kaur P. (2017). Ethosomes and Transfersomes: Principles, Perspectives and Practices. Curr. Drug Deliv..

[11] Song C.K., Balakrishnan P., Shim C.-K., Chung S.-J., Chong S., Kim D.-D. (2012). A novel vesicular carrier, transethosome, for enhanced skin delivery of voriconazole: Characterization and in vitro/in vivo evaluation. Colloids Surf. B Biointerfaces.

[12] Fernández-García R., Lalatsa A., Statts L., Bolás-Fernández F., Ballesteros M.P., Serrano D.R. (2020). Transferosomes as nanocarriers for drugs across the skin: Quality by design from lab to industrial scale. Int. J. Pharm..

[13] Chen Z., Li B., Liu T., Wang X., Zhu Y., Wang L., Wang X., Niu X., Xiao Y., Sun Q. (2017). Evaluation of paeonol-loaded transethosomes as transdermal delivery carriers. Eur. J. Pharm. Sci..

[14] Sguizzato M., Ferrara F., Hallan S.S., Baldisserotto A., Drechsler M., Malatesta M., Costanzo M., Cortesi R., Puglia C., Valacchi G. (2021). Ethosomes and transethosomes for mangiferin transdermal delivery. Antioxidants.

[15] Esposito E., Calderan L., Galvan A., Cappellozza E., Drechsler M., Mariani P., Pepe A., Sguizzato M., Vigato E., Dalla Pozza E. (2022). Ex vivo evaluation of ethosomes and transethosomes applied on human skin: A comparative study. Int. J. Mol. Sci..

[16] Ferrara F., Benedusi M., Sguizzato M., Cortesi R., Baldisserotto A., Buzzi R., Valacchi G., Esposito E. (2022). Ethosomes and transethosomes as cutaneous delivery systems for quercetin: A preliminary study on melanoma cells. Pharmaceutics.

[17] Abdulbaqi I.M., Darwis Y., Assi R.A., Khan N.A.K. (2018). Transethosomal gels as carriers for the transdermal delivery of colchicine: Statistical optimization, characterization, and ex vivo evaluation. Drug Des..

[18] Seenivasan R., Halagali P., Nayak D., Tippavajhala V.K. (2025). Transethosomes: A Comprehensive Review of Ultra-Deformable Vesicular Systems for Enhanced Transdermal Drug Delivery. AAPS PharmSciTech.

[19] Ascenso A., Raposo S., Batista C., Cardoso P., Mendes T., Praça F.G., Bentley M.V.L.B., Simões S. (2015). Development, characterization, and skin delivery studies of related ultradeformable vesicles: Transfersomes, ethosomes, and transethosomes. Int. J. Nanomed..

[20] Samari M., Kashanian S., Zinadini S., Derakhshankhah H. (2025). Enhanced delivery of azithromycin using asymmetric polyethersulfone membrane modified with KIT-6 mesoporous material: Optimization and mechanistic studies. Eur. J. Pharm. Sci..

[21] Timoumi S., Mangin D., Peczalski R., Zagrouba F., Andrieu J. (2014). Stability and thermophysical properties of azithromycin dihydrate. Arab. J. Chem..

[22] Dhoble P., Tekade B., Gorde N., Bodke V. (2024). Solubility Enhancement of BCS Class II Antibiotic (Azithromycin) by Solid Dispersion Technique by Using Skimmed Milk. IJDDT.

[23] Phatale V., Vaiphei K.K., Jha S., Patil D., Agrawal M., Alexander A. (2022). Overcoming skin barriers through advanced transdermal drug delivery approaches. J. Control. Release.

[24] Stulberg D.L., Penrod M.A., Blatny R.A. (2002). Common bacterial skin infections. Am. Fam. Physician.

[25] Pillsbury D.M., Rebell G. (1952). The bacterial flora of the skin. J. Invest. Dermatol..

[26] Ray P., Singh S., Gupta S. (2019). Topical antimicrobial therapy: Current status and challenges. Indian J. Med. Microbiol..

[27] Eleraky N.E., Allam A., Hassan S.B., Omar M.M.J.P. (2020). Nanomedicine fight against antibacterial resistance: An overview of the recent pharmaceutical innovations. Pharmaceutics.

[28] Zou W., McAdorey A., Yan H., Chen W. (2023). Nanomedicine to overcome antimicrobial resistance: Challenges and prospects. Nanomedicine.

[29] Wang S., Gao Y., Jin Q., Ji J. (2020). Emerging antibacterial nanomedicine for enhanced antibiotic therapy. Biomater. Sci..

[30] McFarland J.W., Berger C.M., Froshauer S.A., Hayashi S.F., Hecker S.J., Jaynes B.H., Jefson M.R., Kamicker B.J., Lipinski C.A., Lundy K.M. (1997). Quantitative structure—activity relationships among macrolide antibacterial agents: In vitro and in vivo potency against Pasteurella multocida. J. Med. Chem..

[31] Rukavina Z., Klarić M.Š., Filipović-Grčić J., Lovrić J., Vanić Ž. (2018). Azithromycin-loaded liposomes for enhanced topical treatment of methicillin-resistant Staphyloccocus aureus (MRSA) infections. Int. J. Pharm..

[32] Saddik M.S., Elsayed M.M., El-Mokhtar M.A., Sedky H., Abdel-Aleem J.A., Abu-Dief A.M., Al-Hakkani M.F., Hussein H.L., Al-Shelkamy S.A., Meligy F.Y.J.P. (2022). Tailoring of novel azithromycin-loaded zinc oxide nanoparticles for wound healing. Pharmaceutics.

[33] Abruzzo A., Parolin C., Rossi M., Vitali B., Cappadone C., Bigucci F.J.A. (2022). Development and characterization of azithromycin-loaded microemulsions: A promising tool for the treatment of bacterial skin infections. Antibiotics.

[34] Khalifa N.E., Abdallah M.H., Elghamry H.A., Khojali W.M., Khafagy E.-S., El-Sayed El-Horany H., Shawky S. (2023). Development of Tea Tree Oil Based Nanoemulgel Loaded with Azithromycin for Enhancing the Antibacterial Activity. Processes.

[35] Munir M., Zaman M., Waqar M.A., Hameed H., Riaz T. (2024). A comprehensive review on transethosomes as a novel vesicular approach for drug delivery through transdermal route. J. Liposome Res..

[36] Veer P.J., Mastiholimath V.S. (2023). Formulation, Characterization, and Optimization of Transethosomes for Enhanced Transdermal Delivery of Methotrexate. J. Pharm. Innov..

[37] Garg V., Singh H., Bhatia A., Raza K., Singh S.K., Singh B., Beg S. (2017). Systematic development of transethosomal gel system of piroxicam: Formulation optimization, in vitro evaluation, and ex vivo assessment. AAPS Pharmscitech.

[38] Zhang J.P., Wei Y.H., Zhou Y., Li Y.Q., Wu X.A. (2012). Ethosomes, binary ethosomes and transfersomes of terbinafine hydrochloride: A comparative study. Arch. Pharmacal Res..

[39] Mashaqbeh H., Obaidat R.M., Alsmadi M.M., Pharmacy I. (2024). Solvent-free method for masking the bitter taste of azithromycin dihydrate using supercritical fluid technology. Drug Dev. Ind. Pharm..

[40] Leroy M., Lefèvre T., Pouliot R., Auger M., Laroche G. (2015). Using infrared and Raman microspectroscopies to compare ex vivo involved psoriatic skin with normal human skin. J. Biomed. Opt..

[41] Weerheim A., Ponec M. (2001). Determination of stratum corneum lipid profile by tape stripping in combination with high-performance thin-layer chromatography. Arch. Dermatol. Res..

[42] Wilms E., Trumpie H., Veenendaal W., Touw D. (2005). Quantitative determination of azithromycin in plasma, blood and isolated neutrophils by liquid chromatography using pre-column derivatization with 9-fluorenylmethyloxycarbonyl-chloride and fluorescence detection. J. Chromatogr. B Anal. Technol. Biomed. Life Sci..

[43] Torres-Flores G., Gonzalez-Horta A., Vega-Cantu Y.I., Rodriguez C., Rodriguez-Garcia A. (2020). Preparation and Characterization of Liposomal Everolimus by Thin-Film Hydration Technique. Adv. Polym. Technol..

[44] El-Sonbaty M.M., Akl M.A., Khalid M., Kassem A.A. (2022). Does the technical methodology influence the quality attributes and the potential of skin permeation of Luliconazole loaded transethosomes?. J. Drug Deliv..

[45] Pardeike J., Schwabe K., Müller R.H. (2010). Influence of nanostructured lipid carriers (NLC) on the physical properties of the Cutanova Nanorepair Q10 cream and the in vivo skin hydration effect. Int. J. Pharm..

[46] Allam A.A., Fathalla D., Safwat M.A., Soliman G.M. (2022). Transferosomes versus transethosomes for the dermal delivery for minoxidil: Preparation and in vitro/ex vivo appraisal. J. Drug Deliv. Sci. Technol..

[47] Bin Jardan Y.A., Ahad A., Raish M., Al-Jenoobi F.I. (2023). Preparation and Characterization of Transethosome Formulation for the Enhanced Delivery of Sinapic Acid. Pharmaceutics.

[48] Gillet A., Compère P., Lecomte F., Hubert P., Ducat E., Evrard B., Piel G. (2011). Liposome surface charge influence on skin penetration behaviour. Int. J. Pharm..

[49] Tian Y., Chen L., Zhang W. (2016). Influence of ionic surfactants on the properties of nanoemulsions emulsified by nonionic surfactants span 80/tween 80. J. Dispers. Sci. Technol..

[50] Zhang X., Wu Y., Zhang M., Mao J., Wu Y., Zhang Y., Yao J., Xu C., Guo W., Yu B. (2017). Sodium cholate-enhanced polymeric micelle system for tumor-targeting delivery of paclitaxel. Int. J. Nanomed..

[51] Cattani V.B., Fiel L.A., Jäger A., Jäger E., Colomé L.M., Uchoa F., Stefani V., Dalla Costa T., Guterres S.S., Pohlmann A.R. (2010). Lipid-core nanocapsules restrained the indomethacin ethyl ester hydrolysis in the gastrointestinal lumen and wall acting as mucoadhesive reservoirs. Eur. J. Pharm. Sci..

[52] Tasi L.-M., Liu D.-Z., Chen W.-Y.J.C., Physicochemical S.A., Aspects E. (2003). Microcalorimetric investigation of the interaction of polysorbate surfactants with unilamellar phosphatidylcholines liposomes. Colloids Surf. A Physicochem. Eng. Asp..

[53] Aodah A.H., Hashmi S., Akhtar N., Ullah Z., Zafar A., Zaki R.M., Khan S., Ansari M.J., Jawaid T., Alam A. (2023). Formulation development, optimization by box–behnken design, and in vitro and ex vivo characterization of hexatriacontane-loaded transethosomal gel for antimicrobial treatment for skin infections. Gels.

[54] Moolakkadath T., Aqil M., Ahad A., Imam S.S., Iqbal B., Sultana Y., Mujeeb M., Iqbal Z. (2018). Development of transethosomes formulation for dermal fisetin delivery: Box–Behnken design, optimization, in vitro skin penetration, vesicles–skin interaction and dermatokinetic studies. Artif. Cells Nanomed. Biotechnol..

[55] Gandhi R., Pillai O., Thilagavathi R., Gopalakrishnan B., Kaul C.L., Panchagnula R. (2002). Characterization of azithromycin hydrates. Eur. J. Pharm. Sci. Off. J. Eur. Fed. Pharm. Sci..

[56] Kumar L., Utreja P. (2020). Formulation and characterization of transethosomes for enhanced transdermal delivery of propranolol hydrochloride. Micro Nanosyst..

[57] Mishra K.K., Kaur C.D. (2022). Screening of process variables using Box–Behnken design in the fabrication of berberine hydrochloride-loaded transethosomes for enhanced transdermal delivery. Thai J. Pharm. Sci..

[58] Du Plessis J., Ramachandran C., Weiner N., Müller D. (1996). The influence of lipid composition and lamellarity of liposomes on the physical stability of liposomes upon storage. Int. J. Pharm..

[59] Matharoo N., Mohd H., Michniak-Kohn B. (2024). Transferosomes as a transdermal drug delivery system: Dermal kinetics and recent developments. Wiley Interdiscip. Rev.: Nanomed. Nanobiotechnol..

[60] Godin B., Touitou E., Rubinstein E., Athamna A., Athamna M. (2005). A new approach for treatment of deep skin infections by an ethosomal antibiotic preparation: An in vivo study. J. Antimicrob. Chemother..

[61] Miatmoko A., Marufah N.A., Nada Q., Rosita N., Erawati T., Susanto J., Purwantari K.E., Nurkanto A., Purwati, Soeratri W. (2022). The effect of surfactant type on characteristics, skin penetration and anti-aging effectiveness of transfersomes containing amniotic mesenchymal stem cells metabolite products in UV-aging induced mice. Drug Deliv..

[62] Adnan M., Haider M.F., Naseem N., Haider T. (2023). Transethosomes: A promising challenge for topical delivery short title: Transethosomes for topical delivery. Drug Res..

[63] Boncheva M., Damien F., Normand V. (2008). Molecular organization of the lipid matrix in intact Stratum corneum using ATR-FTIR spectroscopy. Biochim. Et Biophys. Acta (BBA)-Biomembr..

[64] Zi M., Ke J., Jiang S., Cui X., Zhang J., Yuan S., Huang S., Wang J., Liu H., Zhang J. (2024). Colchicine-loaded transethosomes enhances transdermal permeability and therapeutic effects of acute gouty arthritis via vesicle extrusion and lipid perturbation. Colloids Surf. A Physicochem. Eng. Asp..

[65] Solleti V.S., Alhariri M., Halwani M., Omri A. (2015). Antimicrobial properties of liposomal azithromycin for Pseudomonas infections in cystic fibrosis patients. J. Antimicrob. Chemother..

[66] Oh Y.-K., Nix D.E., Straubinger R.M. (1995). Formulation and efficacy of liposome-encapsulated antibiotics for therapy of intracellular Mycobacterium avium infection. Antimicrob. Agents Chemother..

[67] Maier A., Ovesen J.L., Allen C.L., York R.G., Gadagbui B.K., Kirman C.R., Poet T., Quinones-Rivera A. (2015). Safety assessment for ethanol-based topical antiseptic use by health care workers: Evaluation of developmental toxicity potential. Regul. Toxicol. Pharmacol..

[68] Ahad A., Al-Saleh A.A., Al-Mohizea A.M., Al-Jenoobi F.I., Raish M., Yassin A.E.B., Alam M.A. (2018). Formulation and characterization of Phospholipon 90 G and tween 80 based transfersomes for transdermal delivery of eprosartan mesylate. Pharm. Dev. Technol..

